# Genome-Wide Analysis of HSP70s in Hexaploid Wheat: Tandem Duplication, Heat Response, and Regulation

**DOI:** 10.3390/cells11050818

**Published:** 2022-02-26

**Authors:** Yunze Lu, Peng Zhao, Aihua Zhang, Junzhe Wang, Mingran Ha

**Affiliations:** 1 Soil Pollution and Ecological Restoration Center, School of Landscape and Ecological Engineering, Hebei University of Engineering, Handan 056038, China; zaihua2018@163.com (A.Z.); hamingran@163.com (M.H.); 2 State Key Laboratory of Crop Stress Biology for Arid Areas, College of Agronomy, Northwest A&F University, Yangling 712100, China; pengzhao@nwsuaf.edu.cn (P.Z.); wangjunzhe@nwsuaf.edu.cn (J.W.)

**Keywords:** hexaploid wheat, *HSP70*, tandem duplication, regulation, heat adaptation

## Abstract

*HSP70s* play crucial roles in plant growth and development, as well as in stress response. Knowledge of the distribution and heat response of *HSP70s* is important to understand heat adaptation and facilitate thermotolerance improvement in wheat. In this study, we comprehensively analyzed the distribution of *HSP70s* in hexaploid wheat (*TaHSP70s*) and its relatives, and we found an obvious expansion of *TaHSP70s* in the D genome of hexaploid wheat. Meanwhile, a large portion of tandem duplication events occurred in hexaploid wheat. Among the 84 identified *TaHSP70s*, more than 64% were present as homeologs. The expression profiles of *TaHSP70s* in triads tended to be expressed more in non-stressful and heat stress conditions. Intriguingly, many *TaHSP70s* were especially heat responsive. Tandem duplicated *TaHSP70s* also participated in heat response and growth development. Further HSE analysis revealed divergent distribution of HSEs in the promoter regions of *TaHSP70* homeologs, which suggested a distinct heat regulatory mechanism. Our results indicated that the heat response of *TaHSP70s* may experience a different regulation, and this regulation, together with the expression of tandem duplicated *TaHSP70s*, may help hexaploid wheat to adapt to heat conditions.

## 1. Introduction

Hexaploid wheat (*Triticum aestivum* L.) is a worldwide staple food crop. In recent years, due to global warming and more frequent extreme high temperature disasters, heat stress has become a major limiting factor that can significantly constrain wheat yield and quality [1,2]. For example, dry, hot wind often occurs in the major wheat growing regions in China and reduces grain yield by 5%–20% [3].

Due to its sessile nature, hexaploid wheat has evolved an elaborate system for adapting to heat stress. Extensive efforts have been made to explain the underlying mechanism of wheat heat adaptation; however, knowledge about wheat heat adaptation is still limited [4,5,6,7,8]. In plants, 70 kDa heat shock proteins (HSP70s) are essential molecular chaperones that function in protein quality control and play a crucial role in regulating heat responsive genes during plant heat response [9,10,11,12,13]. Heat shock transcription factor A1s, the important regulator of plant heat response, is tightly regulated by the HSP70s/HSP90s complex [14,15]. In addition, many proteins including other HSPs (e.g., HSP100s and small HSPs) have been identified as the substrates or cooperators of HSP70s in mediating plant heat response [9,12,13,16]. Thus, plant heat tolerance is thought to be mainly dependent on HSP70s, possibly due to their master role in plant protein homeostasis [17,18].

Genome-wide analysis and expression patterns of *HSP70s* have been determined in many plant species, improving the understanding of this gene family regarding plant growth and stress response [19,20,21,22]. In Arabidopsis and rice, first, the HSP70 superfamily was divided into the DnaK (HSP70 subfamily) and SSE (HSP110 subfamily) families. The DnaK family members were further assigned to the subfamilies cytosol, BiP, plastid, mitochondria, and T, according to their subcellular localization predicted by the presence of consensus sequences [20,22,23]. A previous genome-wide analysis found that the hexaploid wheat genome contained 114 HSP70s [21]. However, the heat expression and regulation of *HSP70s*, the potentially dominant chaperones in plant heat response, are still largely unknown in hexaploid wheat.

Hexaploid wheat presents three subgenomes (AA, BB, DD) which evolved from two major interspecies hybridizations. The first hybridization occurred between diploid *T. Urartu* (AA progenitor) and a diploid Aegilops species (possibly *Aegilops sharonensis* or a closed species, BB progenitor) and resulted in the formation of allotetraploid wild emmer wheat (*T. turgidum* ssp. *dicoccoides*, AABB). The second hybridization occurred between tetraploid emmer wheat and the diploid *Ae. tauschii* (DD progenitor) and led to the emergence of hexaploid wheat (AABBDD) [24,25]. During the evolution of hexaploid wheat, gene loss or retention and gene gain events occurred within subgenomes, and finally, about 35.8% of the whole genes were evenly distributed throughout the three subgenomes (1:1:1). The three homeologs with a single copy at each homeologous locus were regarded as a triad [26]. The dynamic evolution of *HSP70s* in hexaploid wheat, however, was still unknown.

The expression and regulation of homeologous genes in hexaploid wheat has been investigated and different ratios of homeologous genes with expression portioning have been found to enable hexaploid wheat to adapt to environmental stimuli such as heat, drought, and salt stress [27,28]. However, whether or not *HSP70s* underwent expression portioning and how the expressions were regulated in hexaploid wheat under heat stress were unclear. Heat shock elements (HSEs) are the crucial cis elements in regulating the expressions of HSPs in heat conditions [29,30,31,32]. The canonical HSEs comprised at least three consecutive pentanucleotide motifs of 5′-nGAAn-3′ (or 5′-nTTCn-3′) [29,30,31]. Nevertheless, varied types of HSEs, including sequence variations and different numbers of pentanucleotide motifs, were characterized, and together with other factors, such as positions of HSEs in the gene promoter region, were found to influence the magnitude of the downstream gene expression [31,33,34].

In this study, we comprehensively analyzed the distribution of *HSP70s* in hexaploid wheat and its relatives and revealed subfamily-specific expansion in the D subgenome due to tandem duplication events. More than 64% of the *TaHSP70s* presented as triads and most of these triads showed balanced expression patterns during wheat growth and development; however, more triads were differentially expressed under heat stress. Subsequent characterization of HSEs indicated that the regulation of these homeologs was possibly divergent. These results improve our knowledge regarding the function and regulation of *TaHSP70s* in hexaploid wheat.

## 2. Results

### 2.1. Subfamily-Specific Expansion of TaHSP70s in D Subgenome

**Figure 1:**
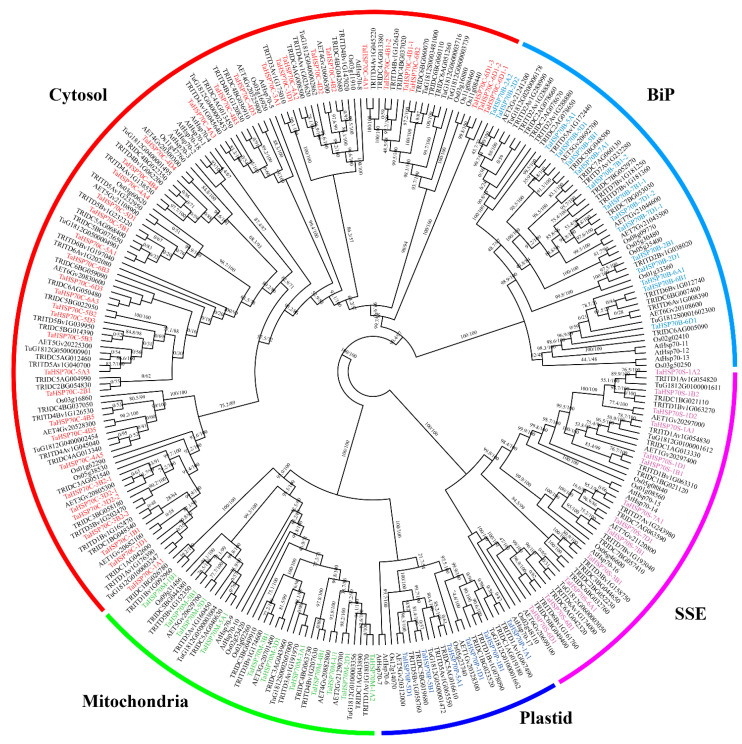
Maximum likelihood phylogenetic analysis of HSP70 proteins. HSP70s from Arabidopsis (At), rice (Os), *T. urartu* (Tu), *Ae. tauschii* (AET), wild emmer wheat (TRIDC), durum wheat (TRITD), and hexaploid wheat (Ta) were used for analysis. Values near branches represent the ultrafast bootstraps as well as the Shimodaira–Hasegawa approximate likelihood ratio test values. TaHSP70s in different subfamilies are shown with different colors.

Using sequence search and motif analysis, totals of 20, 24, 56, 49, and 84 *HSP70s* were characterized in *T. urartu*, *Ae. tauschii*, wild emmer wheat, durum wheat, and hexaploid wheat, respectively. In addition, these *HSP70s* were clearly divided into subfamilies cytosol, BiP, mitochondria, plastid, and SSE, based on the phylogenetic tree (Figure 1, Figure S1, and Table S1). In each subfamily, many subclades were also characterized, and in most subclades, the gene phylogeny was roughly consistent with the species phylogeny, particularly among the *Triticeae* species.

**Figure 2:**
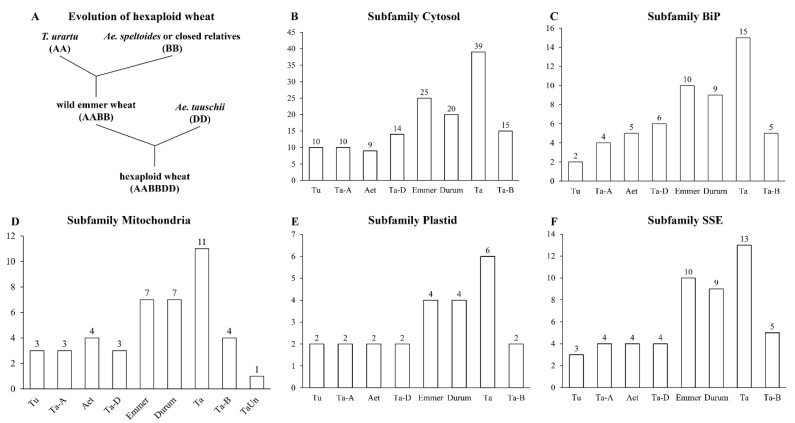
Distribution of *HSP70s* in wheat relatives: (**A**) evolution model of hexaploid wheat; (**B**–**F**) number of *HSP70s* in different subfamilies in different species and subgenomes. Aet, *Ae. Tauschii*; Ta, hexaploid wheat; Emmer, wild emmer wheat; Durum, durum wheat; Tu, *T.urartu*; Ta-A, A subgenome of hexaploid wheat; Ta-B, B subgenome of hexaploid wheat; Ta-D, D subgenome of hexaploid wheat.

The relatively clear polyploidization process of hexaploid wheat made it feasible to study the evolution of *HSP70s* in wheat and its progenitors (Figure 2A). Due to the incomplete genome sequences of the B progenitors, characterization of *HSP70s* in these species was not performed. The number of *HSP70s* was about 20 in diploid progenitors, and about 50 in tetraploid species. In addition, the number of *HSP70s* in hexaploid wheat (84) was not the sum of that in *Ae. tauschii* (24) and wild emmer wheat (56). We further analyzed the number of *HSP70s* in each subfamily (Figure 2B–F). Interestingly, the number of *HSP70s* in hexaploid wheat was similar to the sum of *HSP70s* in *Ae. tauschii* and wild emmer wheat, except for the subfamily cytosol. In the subfamily cytosol, the number of *TaHSP70s* in the A and B subgenomes was equal to the number in wild emmer wheat, while an obvious distinction was found between the number of *HSP70s* in hexaploid wheat D subgenome (13) and *Ae. tauschii* (8).

**Figure 3:**
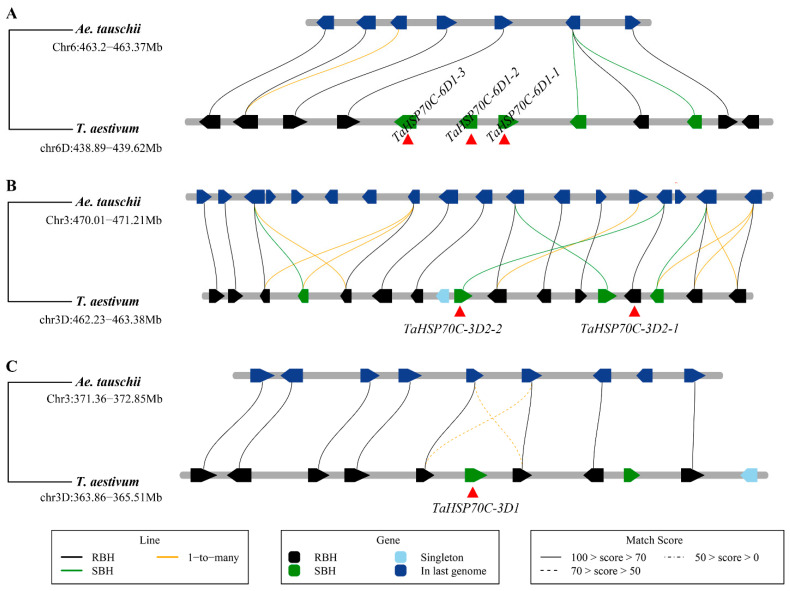
Synteny analysis of the duplicated *TaHSP70s* in the subfamily cytosol. Genes were represented by pentagons, types of homeologous relationship are indicated by different colors. RBH, reciprocal best hits. SBH, single-side best hits. (**A**–**C**) Distribution of genes next to the duplicated *TaHSP70s* in the collinear blocks between *Ae. tauschii* and hexaploid wheat.

To investigate the reason for the increased number of *TaHSP70s* in the D subgenome, gene duplication events were identified and three tandem duplicated gene couples were found (*TaHSP70C-3D2-1/TaHSP70C-3D2-2*, *TaHSP70C-6D1-1/TaHSP70C-6D1-2*, and *TaHSP70C-6D1-2/TaHSP70C-6D1-3*). A synteny analysis between hexaploid wheat and *Ae. tauschii* confirmed these duplications were hexaploid wheat specific (Figure 3A,B). Another gene, *TaHSP70C-3D1*, was also gained in hexaploid wheat (Figure 3C). Six more tandem duplicated gene couples were also found in the subfamilies cytosol, BiP, and SSE: *TaHSP70C-3B2-1/TaHSP70C-3B2-2*, *TaHSP70B-7B1-1/TaHSP70B-7B1-2*, *TaHSP70B-7D1-1/TaHSP70B-7D1-2*, *TaHSP70S-1A1/TaHSP70S-1A2*, *TaHSP70S-1B1/TaHSP70S-1B2*, and *TaHSP70S-1D1/TaHSP70S-1D2*. The synteny analysis revealed that these six duplications had already occurred in progenitors of hexaploid wheat (Figure S2).

### 2.2. High Ratio of TaHSP70 Homeologs Present as Triads in Hexaploid Wheat Genome

**Figure 4:**
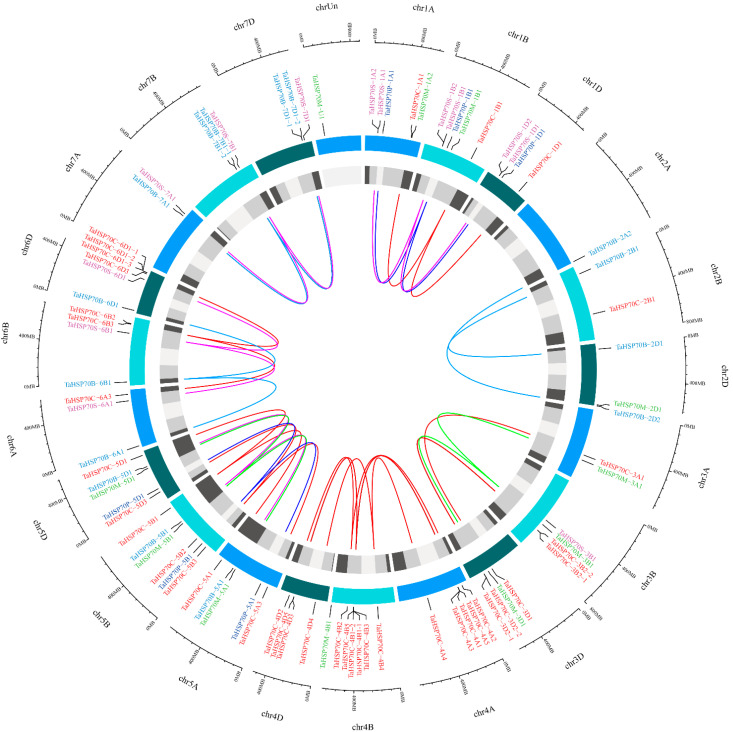
Distribution and homeologous groups of *TaHSP70s* in the wheat genome. The outer track represents chromosomes and the inner track represents chromosome segments. Homeologous TaHSP70s were linked and colored based on subfamilies.

The number of *TaHSP70s* in A, B, and D genomes in each subfamily were not identical, except for subfamily plastid (Figure 2); thus, we analyzed the homeologous groups in each subfamily based on the phylogenetic tree (Figure 4). In the wheat genome, about 35.8% of the genes were present as homeologous groups, and each group consisted of a single gene copy located on the A, B, and D genomes (1:1:1); thus, these groups were regarded as triads [26]. As compared with all genes, the ratio of *TaHSP70s* present as triads was much higher (64.3% vs. 35.8%, Table 1), but the ratio of singletons was much lower (9.5% vs. 37.1%). Homeolog-specific duplication only occurred in subfamily BiP, and loss of one homeolog event occurred in subfamilies cytosol and BiP, singletons largely present in the subfamily mitochondria. In summary, a high ratio of *TaHSP70* homeologs present as triads, and the *TaHSP70* homeologs in subfamily BiP experienced more gene loss/duplication events.

### 2.3. Exon/Intron Structures, Conserved Motifs, and Cis Element Analysis

**Figure 5:**
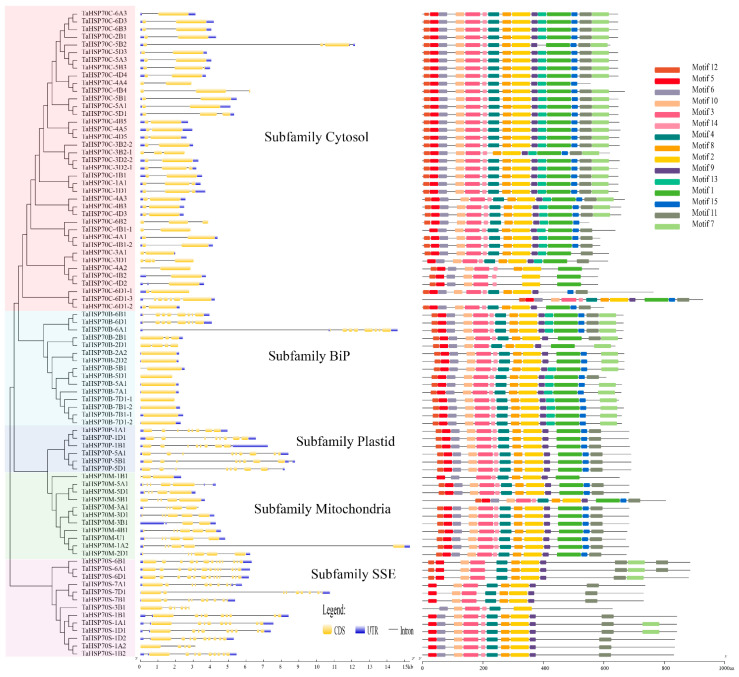
Exon/intron structures, protein motifs of *TaHSP70s*.

The exon/intron structures were also analyzed (Figure 5). The number of exons ranged from 1 to 13, and the average numbers were 2.4, 2.8, 6, 8.5, and 10.2 for subfamilies cytosol, Bip, plastid, mitochondria, and SSE, respectively. The former two subfamilies tended to contain fewer introns. Furthermore, in 14 triads, at least one homeolog showed different exon/intron structures in each triad. These results were quite different from the conserved gene structures and protein motifs of TaHSP90 triads [35].

However, the protein motifs of more than two-thirds of the TaHSP70 triads (13 out of 18) were highly conserved. Intriguingly, the TaHSP70s in subfamilies cytosol and BiP contained similar motifs; TaHSP70s in subfamilies plastid and mitochondria lacked Motifs 7 and 13; TaHSP70s in subfamily SSE were short of Motifs 1, 13, and 15. Surprisingly, although most TaHSP70s in subfamily cytosol contained almost all the motifs, three proteins (TaHSP70C-4A2, TaHSP70C-4B2, and TaHSP70C-4D2) lacked more than 6–7 motifs.

**Figure 6:**
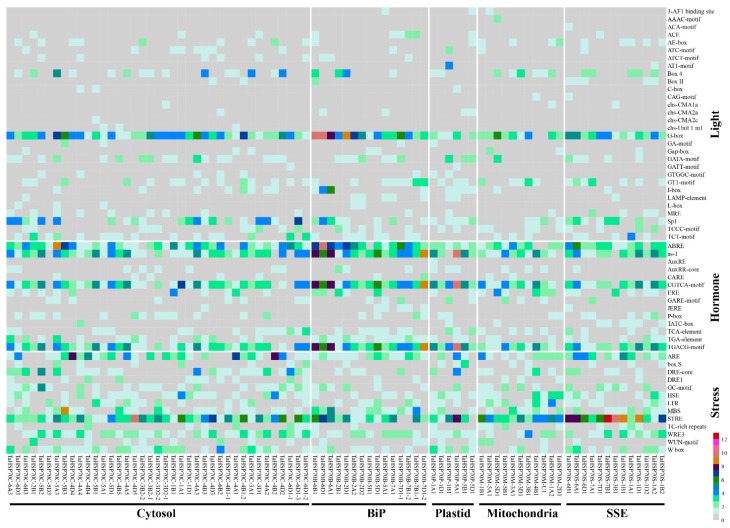
Distribution of cis elements in the promoters of *TaHSP70s*.

Cis elements are important regulatory factors that determine gene transcriptions, thus enable plants to adapt to different environments. The 2 kb genomic sequences upstream from the transcription initiation site of *TaHSP70s* were isolated and subjected to a cis element analysis. In total, 57 cis elements were identified, and different categories of cis elements were distributed in all the 84 *TaHSP70s*, in which cis elements involved in hormone and stress response were more frequent (Figure 6 and Table S2). Elements of G-box, ABRE, as-1, CGTCA, TGACG-motif, ARE, and STRE were predominant in numbers, and these elements were distributed in almost all TaHSP70s. These results imply that *TaHSP70s* play important roles in plant growth and development as well as in stress response.

### 2.4. Expression Patterns of TaHSP70s in Different Tissues and Stages

**Figure 7:**
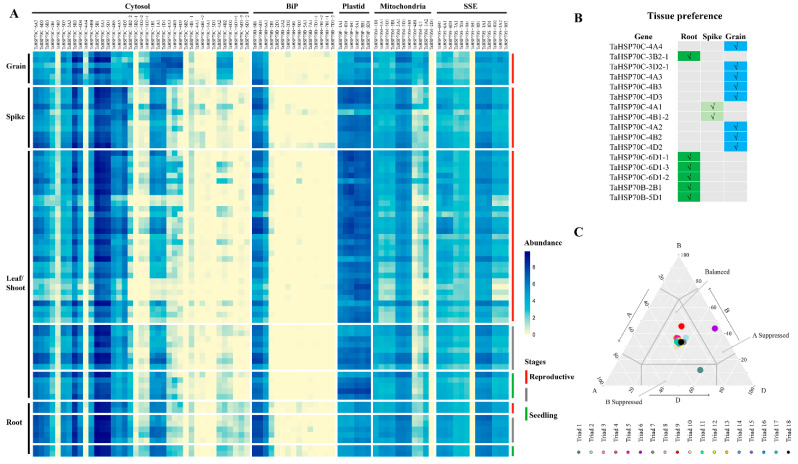
Expression profiles of *TaHSP70s* in wheat variety “Azhurnaya”: (**A**) expression abundance of *TaHSP70s* in different tissues during different developmental stages. Abundance is shown as log2(tpm + 1); (**B**) tissue preference of *TaHSP70s*; (**C**) expression categories of *TaHSP70* triads in all samples.

The spatiotemporal expression profiles of genes can provide useful information for understanding the roles of genes in plant growth and development; thus, we analyzed the expression profiles of *TaHSP70s* in different tissues from the hexaploid wheat variety “Azhurnaya” during different stages [36]. Genes with expression abundance (transcripts per million in unit, tpm) less than 0.5 (tpm <0.5) in all samples were assigned as not expressed; those with a tpm value above 1 in at least one sample were assigned as highly expressed; others were regarded as lowly expressed. Under these criteria, among the 84 *TaHSP70s*, 14 *TaHSP70s* (in which 3, 9, 1, and 1 belonged to subfamilies cytosol, BiP, mitochondria, and SSE, respectively) were not expressed (Figure 7A). Six (evenly distributed in subfamilies cytosol and BiP) out of these 14 *TaHSP70s* were also not expressed in another wheat variety “Chinese Spring” (Figure S3). Furthermore, only three duplicated genes were not expressed, indicating that many duplicated *TaHSP70s* functioned during wheat development. Intriguingly, in the subfamily BiP, 11 of the 15 TaHSP70s were not expressed (average tpm in each tissue < 0.5), three *TaHSP70s* were expressed remarkably higher than in other members, implying their important roles in wheat growth and development.

For the highly expressed genes, the expression level of each gene was normalized by the ratio of average expression abundance in different tissues (root, leaf/shoot, spike, and grain) to the sum of average expression abundance in all tissues, to reveal the expression preference of *TaHSP70s*. Using a threshold of 50% in one tissue type, six, two, and eight *TaHSP70s* were preferred to express in root, spike, and grain, respectively (Figure 7B).

In each subfamily, *TaHSP70* triads, except one triad (including *TaHSP70B-5A1/-B1/-D1*), tended to be more highly expressed than *TaHSP70s* in other types. This phenomenon was also observed in another wheat variety “Chinese Spring” (Figure S3). Thus, expression bias categories of these triads were further analyzed (Figure 7C and Figure S4). In each tissue and across all tissues, 16 out of the 18 triads (account for 88.9%) always showed balanced expression patterns, which were higher than those in all triads in wheat whole genome (72.5% average and 62.6%–78.9% in different tissues). In addition, one triad (including *TaHSP70C-4A4/-B4/-D4*) were always A-suppressed type, another triad (including *TaHSP70B-5A1/-B1/-D1*) were assigned as different patterns possibly due to their low expression abundance (average tpm values were all less than 0.5 in each tissue). Similar expression bias categories of *TaHSP70* triads were also observed in “Chinese Spring” (Figure S5), suggesting that expression patterns of *TaHSP70* homeologs were balanced within triads and conserved across tissues in normal condition.

### 2.5. Expression Patterns of TaHSP70s in Heat Stress Condition

**Figure 8:**
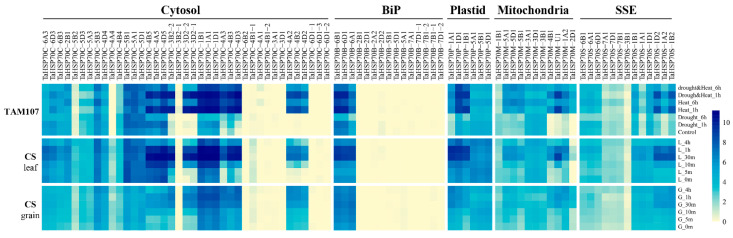
Heat expression profiles of *TaHSP70s* in different wheat varieties. Expression abundance is shown as log_2_(tpm + 1).

*HSP70s* play crucial roles in plant heat response [9]. Using transcriptome data from two wheat varieties, we analyzed the heat expression profiles of *TaHSP70s* (Figure 8). Among all the 84 *TaHSP70s*, 21 and 22 *TaHSP70s* were not expressed (tpm < 0.5 in all samples, including control and stress condition) in wheat variety cv. Chinese Spring, TAM107, of which 20 *TaHSP70s* were shared by both varieties. Thirteen genes were further found to be not transcribed under salt stress, PEG treatment, phosphate starvation, and nitrogen treatment condition (Table S3), implying that these *TaHSP70s* had limited roles in abiotic stress condition. Furthermore, five and seven *TaHSP70s* in leaf samples of Chinese Spring and TAM107, were found not expressed in non-stressful condition but highly expressed in heat stress condition, in which five (*TaHSP70C-3D2-2*, *TaHSP70C-4A2*, *TaHSP70C-4B2*, *TaHSP70C-4D2*, *TaHSP70M-2D1*) were common in both samples.

Further analysis characterized 50 and 42 heat responsive *TaHSP70s* in TAM107 and Chinese Spring (fold change of ≥2, FDR-adjusted *p*-value of <0.01), respectively, of which 40 were common in these two accessions, indicating that the roles of these genes may be conserved (Figure S6). Nine *TaHSP70s* from tandem duplicated gene couples were also heat responsive. In addition, most of the heat responsive *TaHSP70s* originated from triads or singletons. Expression bias categories of triads in different wheat varieties under different conditions were also analyzed, and we found that two to eight triads showed partitioned profiles (Figure S7); thus, more triads underwent differential expression in heat response. Intriguingly, in TAM107, nearly half of the heat responsive *TaHSP70s* in subfamily cytosol also responded to drought or combined heat and drought stress, while all the heat responsive *TaHSP70s* in other subfamilies either uniquely responded to heat stress or were upregulated in heat stress but downregulated in drought stress. The expression patterns of some *TaHSP70s* were confirmed by qRT-PCR in TAM107 and Chinese Spring (Figure S8). These results suggest that those genes may confer different roles in heat and drought conditions.

### 2.6. Distinct HSEs Architecture in the Upstream Regions of TaHSP70s in a Triad

HSEs, which comprise typical consensus sequence ‘NNGAANNTTCNNGAA’ or ‘NNTTCNNGAANNTTC’, are the key cis elements to mediate gene expression in heat condition [32]. Using our previous procedure [33], HSEs were successfully characterized in 47 *TaHSP70s* (Table S4). We further analyzed the relationship between the HSE architecture and heat response pattern in Chinese Spring.

In grains, 24 out of the 47 genes were heat responsive, while in flag leaves this ratio increased to 31:47. Further analysis was conducted to find out why HSE-containing *TaHSP70s* responded to heat stress (Table 2). When considering the number of HSEs, *TaHSP70s* that comprised more than one HSE preferred to be heat responsive (χ^2^ = 3.6947, *p* = 0.0547 in grains and χ^2^ = 4.7435, *p* = 0.0294 in flag leaves). For *TaHSP70s* which contained only one HSE, genes whose HSEs were located less than 500 bp from the transcription start site were more likely to respond to heat (χ^2^ = 6.9942, *p* = 0.0082 in grains and χ^2^ = 17.7397, *p* = 2.53 × 10^−5^ in flag leaves).

As compared with normal condition, more *TaHSP70* triads were differentially expressed in heat stress; thus, we further analyzed the distribution and architecture of HSEs in their promoter regions. Surprisingly, for the number, subunit, type, and position of HSE, at least one factor in one *TaHSP70* homeolog was different from the other two homeologs in a triad. The most distinct situation was found in two triads (*TaHSP70C-1A1, TaHSP70C-1B1, TaHSP70C-1D1; TaHSP70C-4A2, TaHSP70C-4B2, TaHSP70C-4D2*), where the number, subunit, type, and position of HSE in a homeolog were all different from each other (Table S4). These results indicated that the heat regulatory mechanism of *TaHSP70* triads was possibly distinct.

## 3. Discussion

*HSP70s* play crucial roles in plant growth and stress response [9]. In this study, first, we comprehensively analyzed the distribution of *HSP70s* in hexaploid wheat and its relatives and found that a set of tandem duplication events occurred in hexaploid wheat. Then, expression profiles of *TaHSP70s* in heat stress revealed that the heat responsive conservation of *TaHSP70* triads and tandem duplicated *TaHSP70s* may also participate in growth and development, while *TaHSP70* homeologs may be differentially expressed in heat and drought conditions. Further work is required to confirm our findings by looking at TaHSP70 protein levels. Finally, the divergent distribution of HSEs in the promoter regions of *TaHSP70* homeologs in a triad suggested their heat regulatory distinction.

### 3.1. TaHSP70s Were Highly Conserved

In this study, more than 64% of the 84 identified *TaHSP70s* were present as homeologs in a triad—in other words, those *TaHSP70s* distributed 1:1:1 in three homeologous groups. This proportion was much higher than that for all genes at a whole-genome level (35.8%) [26]. A high proportion of gene triads was also found in *TaHSP90s* (100% of 18 genes) [35]. The high percentage of gene triad retention, particularly the key molecular chaperons, may be due to their important roles in plant growth and stress response. Consistently, as compared with other types, *TaHSP70s* in triads tended to be expressed more normally and were the major heat responsive genes in abiotic stress conditions. However, further functional characterizations of these homeologs are needed to confirm their functions and whether to support this hypothesis or not. Examples of expressions of three gene homeologs were very common in hexaploid wheat growth and heat adaptation [28,36]. Retention and functional validation of three gene homeologs has been found in hexaploid wheat *CONSTANS* and *VRNs* [37,38].

### 3.2. Regulatory Mechanism of TaHSP70s May Be Divergent

More triads experienced expression partitioning, which led to the question of whether HSEs, the key heat responsive elements, were conserved or not in the promoter regions of *TaHSP70* homeologs. Further analysis showed the distinct distribution of HSEs in three *TaHSP70* homeologs. The varied architecture of HSEs was found to lead to different heat response magnitudes of the downstream genes in our previous study [33]. The varied HSEs of *TaHSP70* homeologs may confer their different response attitudes in different heat conditions. In addition, variations in promoters have been proven to result in expression partitioning and function alteration of homeologs, such as *wheat LEAFY HULL STERILE1* and *VERNALIZATION 1*, in hexaploid wheat [38,39]. More efforts are needed to characterize the function of each homeolog. Here, we speculate that the regulatory mechanism of *TaHSP70s* may be different and complex.

### 3.3. Tandem Duplicated TaHSP70s May Facilitate Heat Adaptation of Hexaploid Wheat

Tandem duplications of genes contribute to increased members of gene families [40,41]. Consistently, there was an increase in the number of *TaHSP70s* in the D genome as compared with *Ae. tauschii*, the D genome progenitor of hexaploid wheat, which was possibly due to the three tandem duplication events in D genome. Tandem duplications of genes also provide important subfunctionalization and neofunctionalization sources for gene evolution, in addition to available variants for plant adaptation to dramatically changing environments [42,43,44]. Many tandem duplications of genes were transcribed to contribute to the tolerance of hexaploid wheat to salt, drought, heat, and a combination of drought and heat stress [27]. Tandem duplications of *HSP20s* were also expressed in potato under heat stress [45]. Similarly, half of the 10 tandem duplications of *TaHSP70* pairs possibly facilitated heat adaptation of hexaploid wheat.

In conclusion, the varied architecture of HSEs possibly not only diversifies the regulation of *TaHSP70s*, but also together with tandem duplicated TaHSP70s, help hexaploid wheat to adapt to heat conditions.

## 4. Materials and Methods

### 4.1. Characterization of HSP70s in Triticeae Species

Genome and protein sequence genomic annotation files of hexaploid wheat (IWGSC RefSeq v1.1 annotation) were downloaded from URGI (http://wheat-urgi.versailles.inra.fr/, accessed on 21 October 2020), protein sequences of *Ae. tauschii*, *T. urartu*, wild emmer wheat (*T. turgidum* ssp. *dicoccoides*), and durum wheat (*T. turgidum* ssp. *durum*) were from Ensembl Plants (http://plants.ensembl.org/index.html, accessed on 21 October 2020). Using the criteria of identify >50% and an e-value < 1 × 10^−5^ in the blastp program, protein sequences of HSP70 from Arabidopsis and rice were subjected as query sequences to blast against protein sequences of Triticeae species, including *Ae. tauschii*, *T. urartu*, wild emmer wheat, durum wheat, and hexaploid wheat. The hidden Markov model profiles of HSP70 (PF00012) were also scanned against these protein sequences in HMMER 3.0, with an e-value threshold of <1 × 10^−5^. The results of blastp and HMMER 3.0 were merged and subjected to the NCBI-CDD database (https://www.ncbi.nlm.nih.gov/cdd, accessed on 30 October 2020) to examine the presence of the HSP70 domain. Finally, protein sequences with molecular weight >60 kDa and containing the HSP70 domain were regarded as HSP70 proteins [19].

### 4.2. Phylogeny Analysis and Synteny Analysis

Protein sequences of HSP70s from Arabidopsis, rice, *Ae. tauschii*, *T. urartu*, wild emmer wheat, durum wheat, and hexaploid wheat were aligned by MAFFT (v7.45) with “L-INS-i” strategy, then, a phylogenetic tree was constructed by IQ-TREE (v2.0-rc1) and evaluated by Ultrafast bootstraps and a Shimodaira–Hasegawa approximate likelihood ratio test (1000 replicates each), with the following parameters: -m MFP -bb 1000 -alrt 1000 -redo [46,47,48,49]. Homeologs were identified by the phylogeny. The synteny analysis and tandem duplication were characterized by Triticeae Gene-Tribe database [50] and MCScanX [51].

### 4.3. Naming of TaHSP70s

According to the phylogenetic tree, *TaHSP70s* were divided into different subfamilies. For convenience, we renamed all *TaHSP70s* according to subfamilies, chromosome location, and phylogenetic relationship. Each gene name began with “Ta”, the abbreviation for Triticum aestivum, followed by the subfamily name represented by a character, and then the chromosome location. Putative homeologs shared the same gene name except for the chromosome location (e.g., *TaHSP70C-1A1*, *TaHSP70C-1B1*, and *TaHSP70C-1D1*). Genes in the same chromosome belonging to the same subfamily were consecutively numbered (e.g., *TaHSP70C-4A2*, *TaHSP70C-4A3*). Paralogs were named with consecutive numbers following the chromosome location with a dash (*TaHSP70C-6D1-1*, *TaHSP70C-6D1-2*). Homeologous genes that were evenly distributed in the A, B, and D genomes, with a ratio of 1:1:1, were regarded as triads, and triads were named by consecutive numbers.

### 4.4. Gene Structure and Sequence Motif Analysis

Intron/exon information of *TaHSP70s* were extracted from the genome annotation file and submitted to the Gene Structure Display Server (GSDS, http://gsds.cbi.pku.edu.cn/index.php, accessed on 16 November 2020) to display the gene structures. Conserved motifs of protein sequences were analyzed using the MEME program (http://meme-suite.org/tools/meme, accessed on 20 November 2020); maximum number of motifs was set as 15 and optimum motif width ranged from 6 to 200 amino acid residues. Finally, the conserved motifs were shown by TBtools [52].

### 4.5. Cis Element Analysis of TaHSP70s

Upstream regions (2000 bp) of *TaHSP70s* from the transcription start site were extracted. HSEs were analyzed by our previous protocol [33]. Briefly, the pentanucleotide motifs 5′-NGAAN-3′ and 5′-NTTCN-3′ were regarded as a subunit and characterized in the upstream regions of TaHSP70s; the subunit numbers must be at least three, and mismatch on the “G” of 5′-NGAAN-3′ (“C” for 5′-NTTCN-3′) in the first and last subunit was not allowed if the subunit number was three. Other cis elements were analyzed in the PlantCARE database [53].

### 4.6. Expression Analysis of TaHSP70s

Expression levels of *TaHSP70s* in different tissues and stages from the wheat variety “Azhurnaya” and “Chinese Spring” were extracted from a previous study [36]. Other transcriptome data were downloaded from the SRA archive (SRP128236, SRP045409) [28,54] and mapped to Chinese Spring RefSeqv1.1+UTR transcriptome reference by kallisto v0.46.2. The gene expression level was summarized by tximport v1.18.0 and fold changes were assigned by DESeq2 v1.30.1 in R program. The heatmap was drawn by R program with the function “pheatmap”. The expression categories of triads were defined as previously described [36]. Briefly, relative expression of each homeolog in a triad was normalized as the ratio of its average expression (tpm value) in a tissue (or across tissues) to the sum of the average expression of three homeologs in the same tissue (or across tissues). For relative expression across all the tissues, the total expression abundance of a gene was calculated as the average of the average expression abundance in each tissue, rather than a simply geometric mean across all samples, to exclude different numbers of samples in each tissue. The Euclidean distance, from the relative expression of each triad to each of the seven ideal categories listed in [36], was calculated. The expression category for each triad was assigned based on the shortest distance.

Some tandem duplicated *TaHSP70s*, as well as several specifically heat upregulated ones were selected for qRT-PCR validation (Table S5). Plants were cultured as described [28]. Briefly, seeds of two wheat cultivars “Chinese Spring” and “TAM 107” were surface-sterilized and then grown in a growth chamber under normal conditions (22 °C/18 °C, 16/8 h). Ten-day-old seedlings were subjected to heat stress (40 °C), drought stress (20 % m/V PEG-6000), and combined heat and drought stress (40 °C and 20% m/V PEG-6000) for 1 and 6 h. Leaves were sampled for RNA isolation. About 1 μg RNA was used for reverse transcription using the PrimeScriptTM RT reagent Kit with a gDNA Eraser (Perfect Real Time) kit (Takara, Dalian, China), following the kit protocol. QRT-PCR was run on a Thermo Fisher Scientific QuantStudio 3 Real-Time PCR System using the TB Green^®^ Premix Ex Taq™ II (Tli RNaseH Plus) kit (Takara, Dalian, China), following the corresponding protocol.

## Figures and Tables

**Table 1:** Groups of homeologous *HSP70s* in hexaploid wheat.

Homeologous Groups (A:B:D)	Subfamily Cytosol		Subfamily BiP		Subfamily Mitochondria		Subfamily Plastid		Subfamily SSE		Sum		Wheat Total Ratio %
	Groups	Genes	Groups	Genes	Groups	Genes	Groups	Genes	Groups	Genes	Genes	Ratio %	
1:1:1	8	24	2	6	2	6	2	6	4	12	54	64.3	35.8
n:1:1/1:n:1/1:1:n	0	0	1	5	0	0	0	0	0	0	5	6.0	5.7
1:1:0/1:0:1/0:1:1	2	6	2	4	0	0	0	0	0	0	10	11.9	13.2
Other ratios	2	7	0	0	0	0	0	0	0	0	7	8.3	8
Singletons	2	2	0	0	5	5	0	0	1	1	8	9.5	37.1

**Table 2:** Distributions of HSEs in the promoter regions of heat responsive TaHSP70s.

	Grains				Flag Leaves				
	Number of HSE	Number of Genes	HSE < 500 bp	HSE > 500 bp		Number of HSE	Number of Genes	HSE < 500 bp	HSE > 500 bp
HS	1	15	12	3	HS	1	20	17	3
	>1	9				>1	11		
non-HS	1	20	7	17	non-HS	1	15	2	13
	>1	3				>1	1		

HS, heat responsive; non-HS, not heat responsive; 500 bp mean distance from transcription initiation site.

## Data Availability

The study did not report new data, all transcriptome data were downloaded from NCBI, which we had mentioned in “Materials and Methods”.

## Supplementary Materials

The following supporting information can be downloaded at: https://www.mdpi.com/article/10.3390/cells11050818/s1. Figure S1: Raw maximum likelihood phylogenetic analysis of HSP70 proteins, Figure S2: Synteny analysis of the duplicated *TaHSP70s* from subfamilies BiP and SSE in hexaploid wheat and its relatives, Figure S3: Expression abundance of *TaHSP70s* in “Chinese Spring”, Figure S4: Expression categories of *TaHSP70* triads in different tissues in “Azhurnaya” under normal condition, Figure S5: Expression categories of *TaHSP70* triads in different tissues in “Chinese Spring” under normal condition, Figure S6: Fold change of *TaHSP70s* in “TAM107” (top) and “Chinese Spring” (bottom) under abiotic stress conditions. Fold change was showed as log2(fold change), Figure S7: Expression categories of *TaHSP70* triads in different tissues in “TAM107”, “Chinese Spring”, “HD2985”, “HD2329” under abiotic stress, Figure S8: QRT-PCR validation of some *TaHSP70s* in “TAM107” and “Chinese Spring” under abiotic stress condition, Table S1: Detail information of TaHSP70s. Chromosome segment was classified according to IWGSC, 2018, Sciencs, Table S2: Distribution of cis-elements in promoters of TaHSP70s, Table S3: Expression of the 20 unexpressed TaHSP70s under other abiotic stress conditions, Table S4: HSEs in TaHSP70s. Distance was showed as the nearest HSE to transcription iniation site, Table S5: Primers for qRT-PCR validation.
