# Supplementary material for: Genome-Wide Analysis of HSP70s in Hexaploid Wheat: Tandem Duplication, Heat Response, and Regulation
Source: Cells. 2022 Feb 26;11(5):818. doi: 10.3390/cells11050818 (PMC8909476; doi:10.3390/cells11050818)
Supplement: Supplementary file 1 [file cells-11-00818-s001.zip › Supplementary materials.pdf]

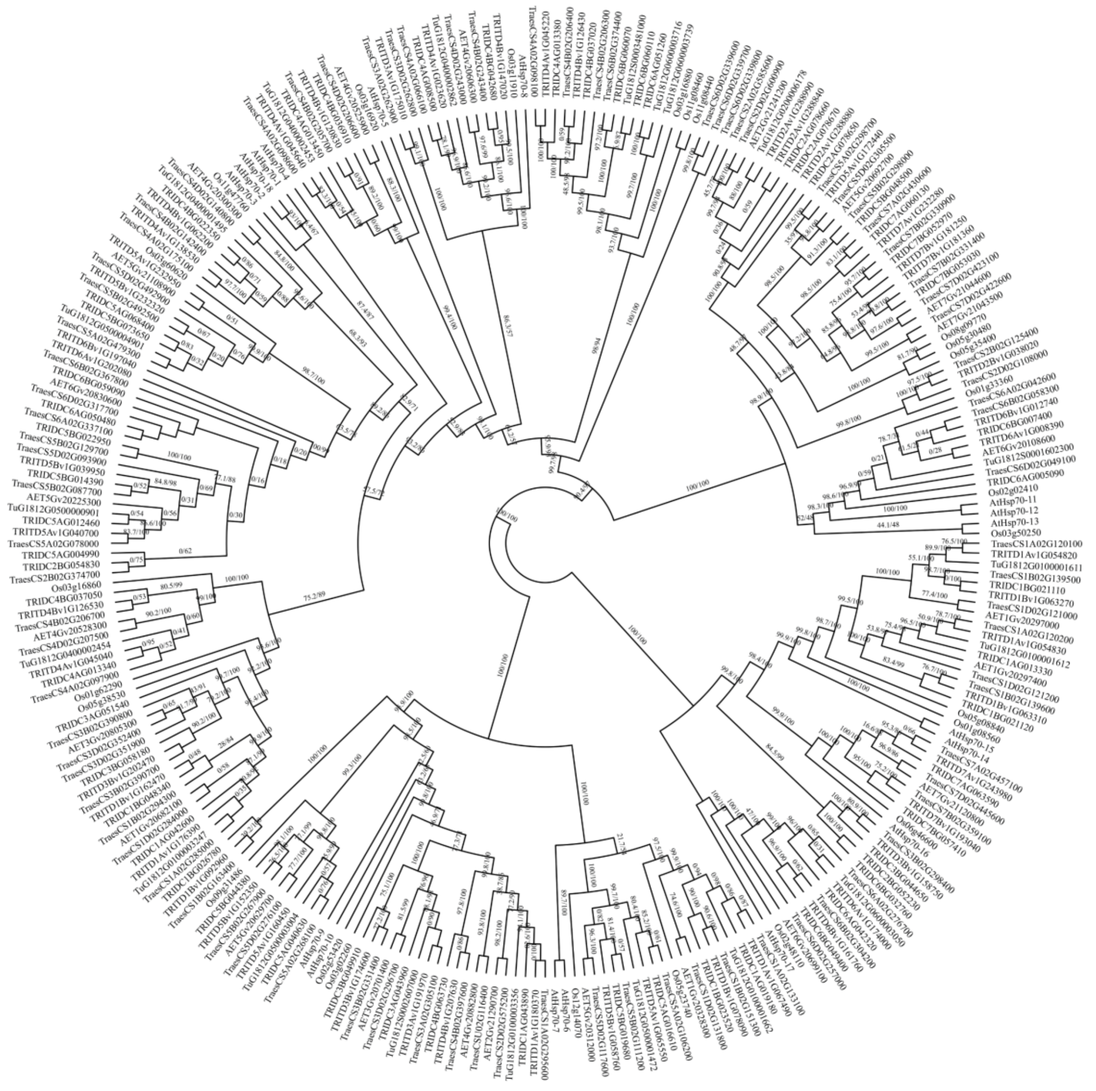

Figure S1. Raw maximum likelihood phylogenetic analysis of HSP70 proteins. HSP70s from Arabidopsis (At), rice (Os), *T. urartu* (Tu), *Ae. tauschii* (AET), wild emmer wheat (TRIDC), durum wheat (TRITD), hexaploid wheat (Ta) were used for analysis. Values near branches represent the Ultrafast bootstraps as well as a Shimodaira-Hasegawa approximate likelihood ratio test values.

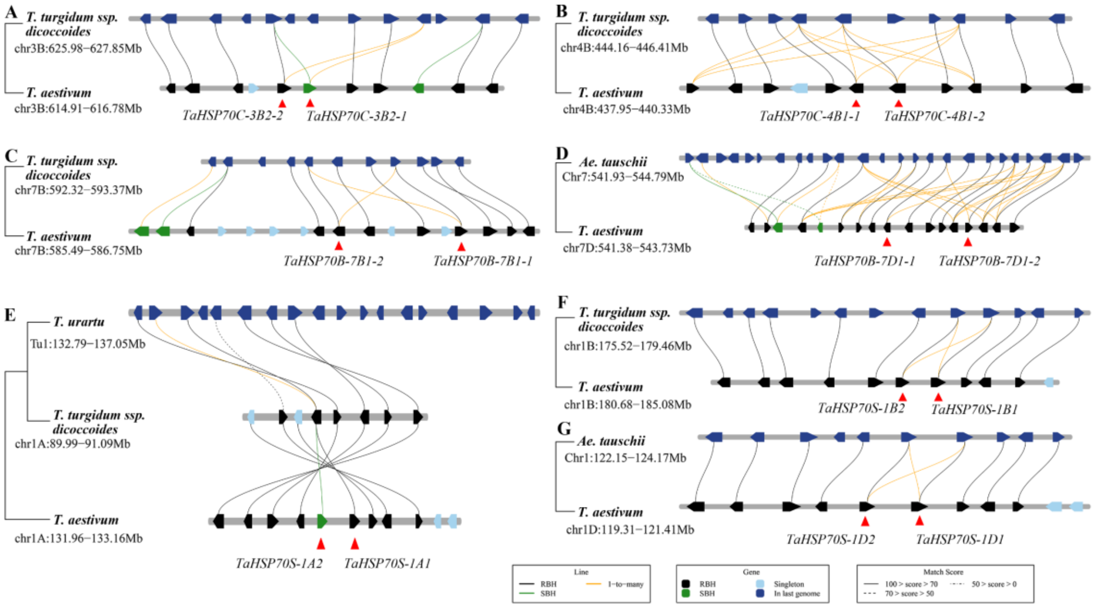

Figure S2. Synteny analysis of the duplicated *TaHSP70s* from subfamilies BiP and SSE in hexaploid wheat and its relatives.

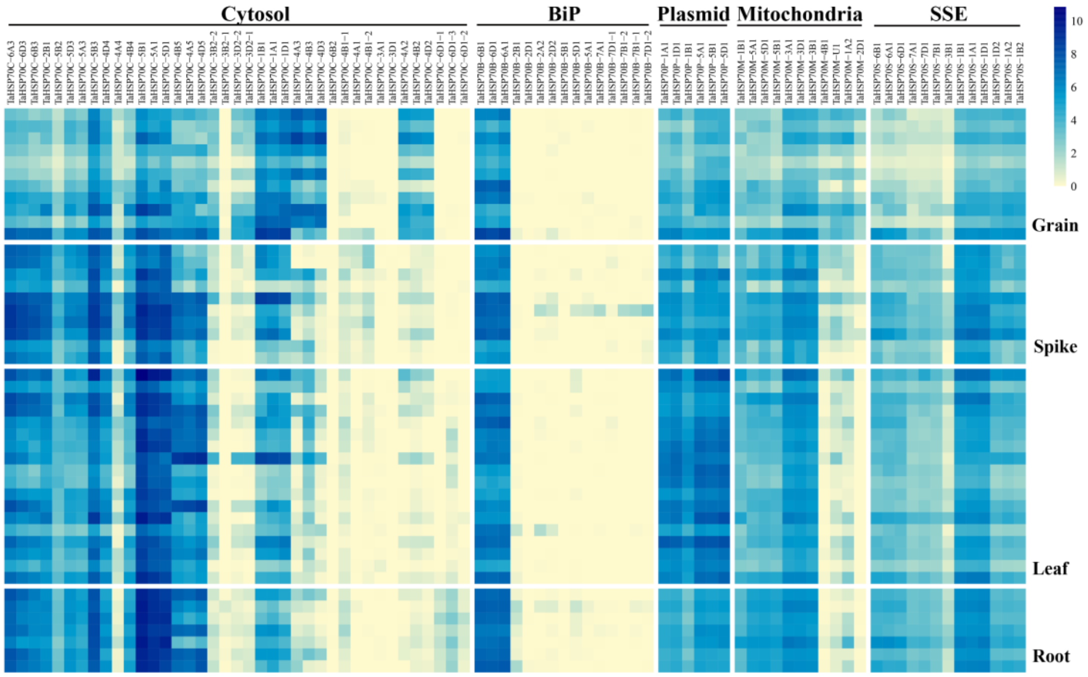

Figure S3. Expression abundance of *TaHSP70s* in "Chinese Spring". Expression abundance was showed as log2(tpm + 1).

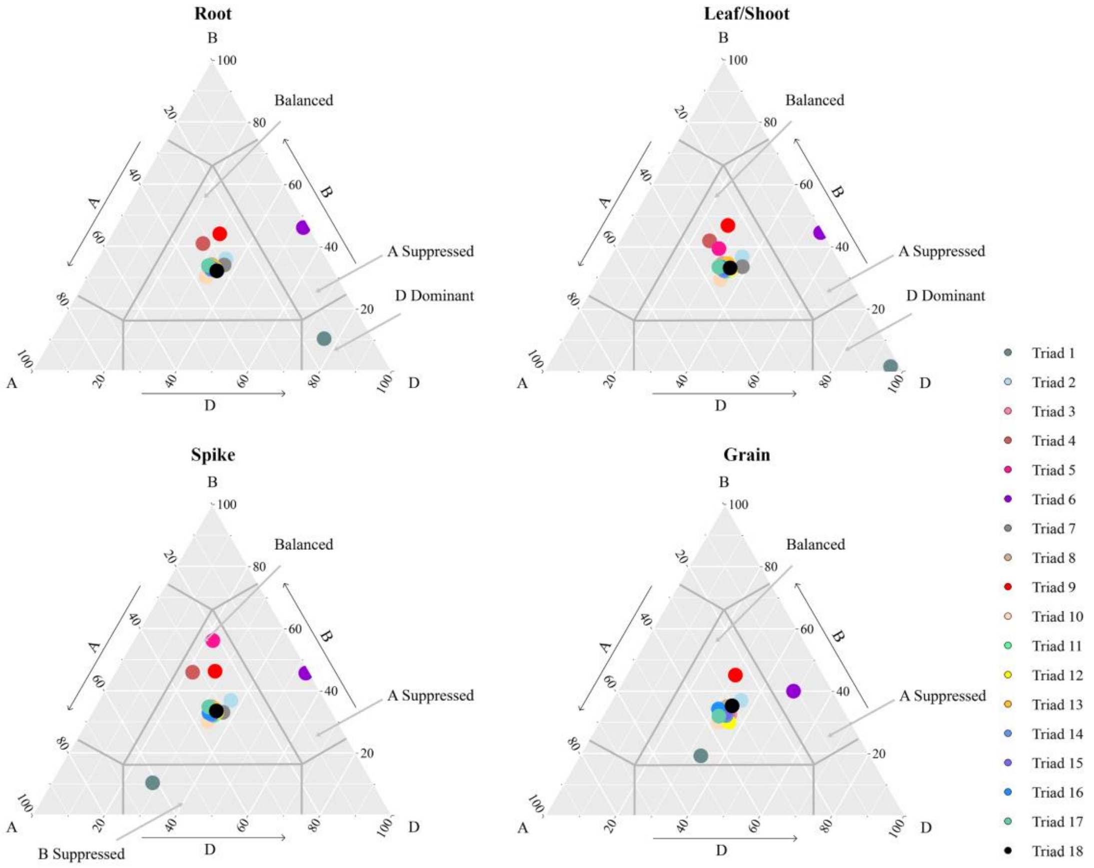

Figure S4. Expression categories of *TaHSP70* triads in different tissues in "Azhurnaya" under normal condition.

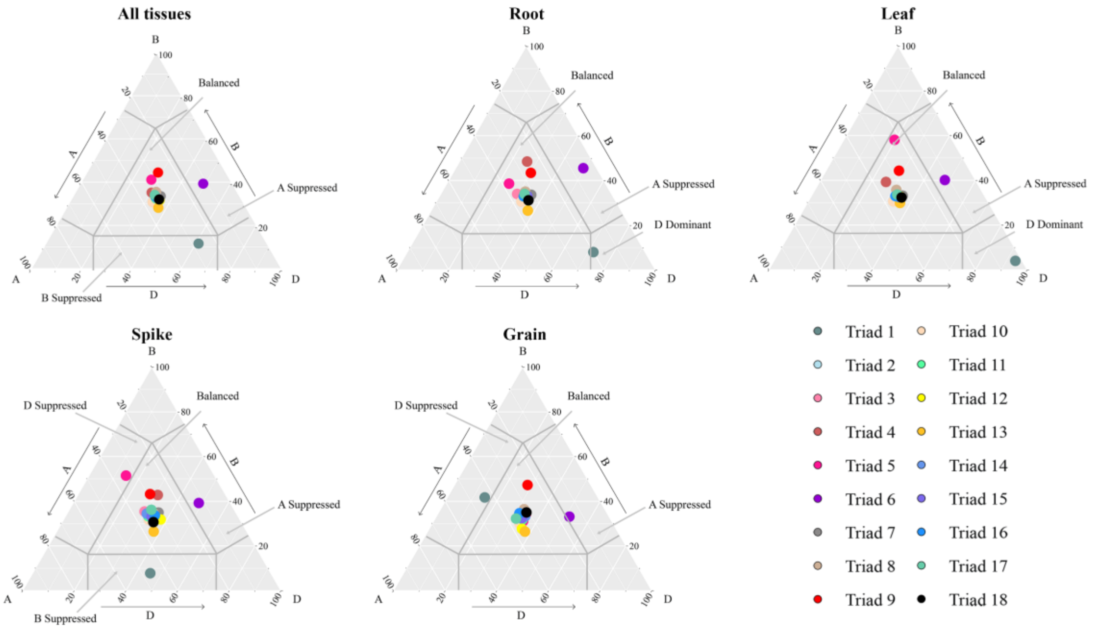

Figure S5. Expression categories of *TaHSP70* triads in different tissues in "Chinese Spring" under normal condition.

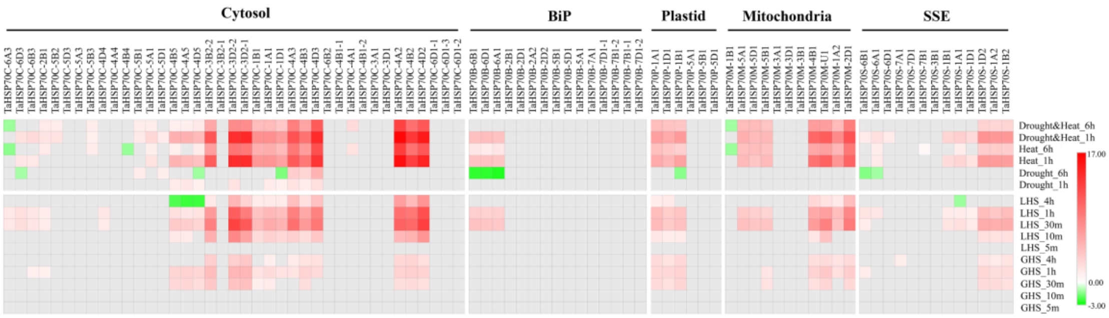

Figure S6. Fold change of *TaHSP70s* in "TAM107" (top) and "Chinese Spring" (bottom) under abiotic stress conditions. Fold change was showed as log2(fold change).

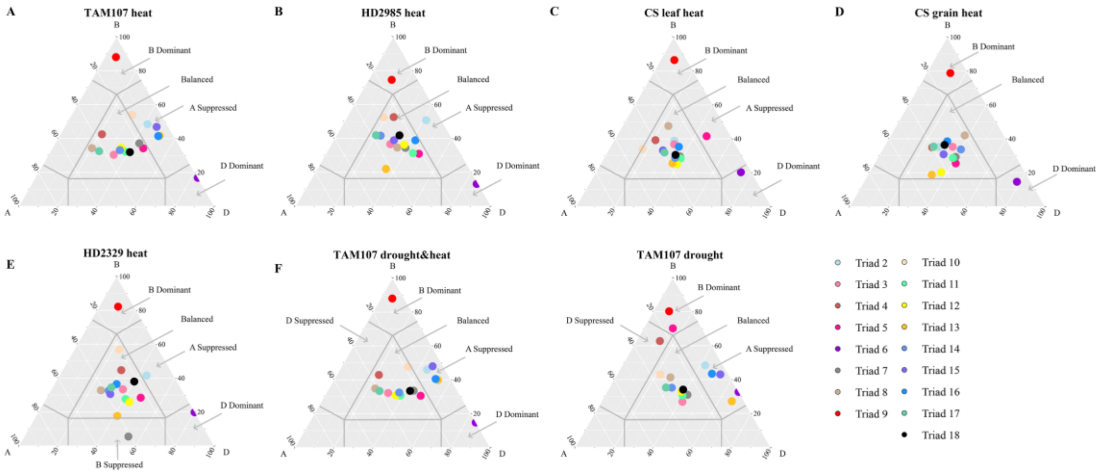

Figure S7. Expression categories of *TaHSP70* triads in different tissues in "TAM107", "Chinese Spring", "HD2985", "HD2329" under abiotic stress.

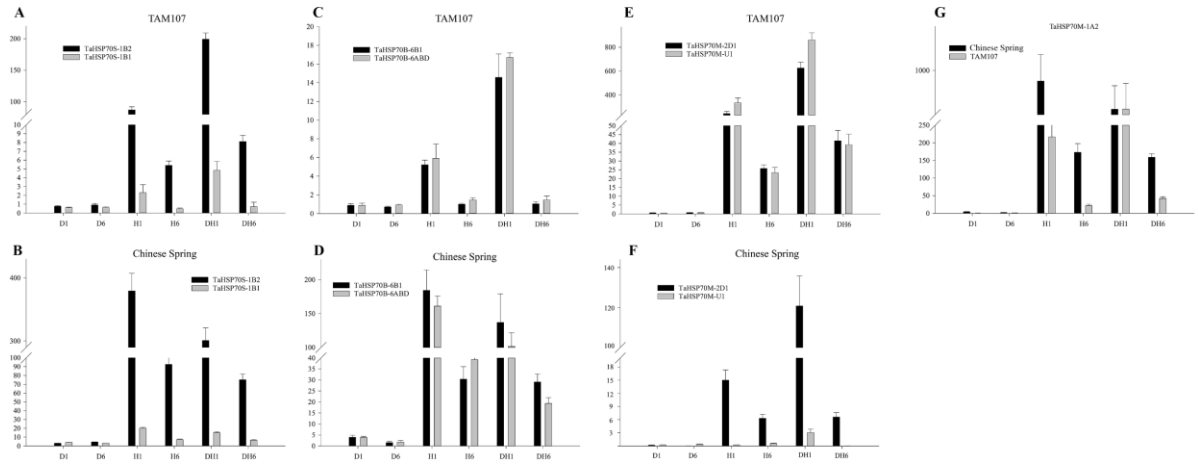

Figure S8. QRT-PCR validation of some *TaHSP70s* in "TAM107" and "Chinese Spring" under abiotic stress condition. X-axis represented conditions: D1, drought stress for 1 hour; D6, drought stress for 6 hours; H1, heat stress for 1 hour; H6, heat stress for 6 hours; DH1, drought and heat stress for 1 hour; DH6, drought and stress for 6 hours. Y-axis showed expression abundance (calculated by $2^{-\Delta\Delta Ct}$), actin was used for control. In (A) and (B), the grey and black bars represent two duplicated TaHSP70s. In (C) and (D), the black bar represented one TaHSP70, the grey bar represented three TaHSP70s (*TaHSP70B-6A*/-*6B*/-*6D*). In (E) and (F), the grey and black bars represent two *TaHSP70s* which were only heat responsive but not drought responsive. In (G), the panel showed expression abundance of *TaHSP70-1A2*, the black and grey bars represented Chinese Spring and TAM107.–
